# Expression of dual Nucleotides/Cysteinyl-Leukotrienes Receptor GPR17 in early trafficking of cardiac stromal cells after myocardial infarction

**DOI:** 10.1111/jcmm.12305

**Published:** 2014-06-07

**Authors:** Simona Cosentino, Laura Castiglioni, Francesca Colazzo, Elena Nobili, Elena Tremoli, Patrizia Rosa, Maria P Abbracchio, Luigi Sironi, Maurizio Pesce

**Affiliations:** a Laboratorio di Biologia e Biochimica dell'Aterotrombosi, Centro Cardiologico Monzino, IRCCSMilan, Italy; b Dipartimento di Scienze Farmacologiche e Biomolecolari, Università di MilanoMilan, Italy; c Dipartimento di Biotecnologie Mediche e Medicina Traslazionale (BIOMETRA), Istituto di NeuroscienzeMilan, Italy; d Laboratorio di Ingegneria Tissutale Cardiovascolare, Centro Cardiologico Monzino, IRCCSMilan, Italy

**Keywords:** myocardial ischaemia, cardiac stromal cell, GPR17, Cysteinyl-Leukotrienes, myofibroblasts

## Abstract

GPR17 is a G_i_-coupled dual receptor activated by uracil-nucleotides and cysteinyl-leukotrienes. These mediators are massively released into hypoxic tissues. In the normal heart, GPR17 expression has been reported. By contrast, its role in myocardial ischaemia has not yet been assessed. In the present report, the expression of GPR17 was investigated in mice before and at early stages after myocardial infarction by using immunofluorescence, flow cytometry and RT-PCR. Before induction of ischaemia, results indicated the presence of the receptor in a population of stromal cells expressing the stem-cell antigen-1 (Sca-1). At early stages after ligation of the coronary artery, the receptor was expressed in Sca-1^+^ cells, and cells stained with Isolectin-B4 and anti-CD45 antibody. GPR17^+^ cells also expressed mesenchymal marker CD44. GPR17 function was investigated *in vitro* in a Sca-1^+^/CD31^−^ cell line derived from normal hearts. These experiments showed a migratory function of the receptor by treatment with UDP-glucose and leukotriene LTD4, two GPR17 pharmacological agonists. The GPR17 function was finally assessed *in vivo* by treating infarcted mice with Cangrelor, a pharmacological receptor antagonist, which, at least in part, inhibited early recruitment of GPR17^+^ and CD45^+^ cells. These findings suggest a regulation of heart-resident mesenchymal cells and blood-borne cellular species recruitment following myocardial infarction, orchestrated by GPR17.

## Introduction

The early response to acute myocardial ischaemia (MI) is dominated by an innate immunity-related process that prevents ventricular rupture through rapid formation of a granulation tissue. This is thereafter replaced by a collagen-abundant scar [1]. During the inflammatory phase, the first trigger for recruitment of leucocytes into the infarcted area is represented by local release of *pro*-inflammatory mediators. Neutrophils and macrophages then clear the wound from dead cells and matrix debris, and produce cytokines/growth factors promoting myofibroblasts recruitment (MFs) [2–4]. The progressive substitution of the contractile myocardium with fibrocellular, Collagen-I enriched and un-contractile tissue results in heart functional alteration; this condition is called post-ischaemic heart failure [3].

Cellular damage associated with tissue hypoxia causes local and systemic release of extracellular nucleotides (*e.g*. ATP/ADP and Uracil-nucleotides UTP/UDP) and Cysteinyl-Leukotrienes (cys-LTs). Extracellular nucleotides have been classically involved in hypoxic cell death [5] or cardioprotection [6,7], whereas Cys-LTs, generated by 5-Lipoxygenase metabolism of Arachidonic acid, play important roles in priming inflammatory signalling in ischaemic tissues [8–11]. In this framework, the P2Y-like GPR17 receptor responds to these ligand families as a dual G_i_-coupled receptor, it is expressed in organs susceptible to ischaemic damage such as the heart, the kidney and the brain [12,13] and it has been recently classified as an oligodendrocyte precursors receptor with a potential function in glial cell differentiation [14,15].

Prompted by a recent appraisal of purinergic signalling in paracrine activity of cardiac stromal cells [16] and lines of evidence showing a complex function of the GPR17 receptor in brain ischaemic damage, in the present contribution, we investigated the expression of the receptor in the mouse heart, before and after MI and partially assessed its function by *in vitro* and *in vivo* studies

## Materials and methods

### Experimental design of the animal model and ethical declaration

Experiments were conducted in accordance with institutional guidelines, conformed to national and international law and policies (4D.L. N.116, G.U., supplement 40, 18-2-1992; EEC Council Directive 86/609, OJ L 358,1,12-12-1987; National Institutes of Health's Guide for the Care and Use of Laboratory Animals and US National Research Council 1996). C57Bl/6N mice (Charles River Laboratories, Calco, Italy), aged 8 weeks (18–20 g bw), were fed *ad libitum* with standard chow/water, and randomly assigned to two groups: sham-operated mice and MI-mice. Surgery and sacrifices were performed under anaesthesia with intraperitoneal 75 mg/kg ketamine cloridrate and 1 mg/kg medetomidine.

### *In vivo* myocardial infarction/pharmacological treatments

Mice were anaesthetized, intubated and ventilated with positive airway pressure. After thoracotomy, MI was induced by permanent ligation of the left anterior descending coronary artery (LAD) as previously reported [17]. Sham-operated mice underwent identical surgical procedure without LAD-ligation. Mice (five animals/group/time-point) were sacrificed at 24 and 48 hrs post-MI for morphological and immunofluorescence (IF) analyses. Further details about surgical procedures, MI quantification, pharmacological treatments, hearts collection and histological processing are provided in the online supplementary material.

### Sca-1^+^ cell line derivation and high-throughput cell sorting from infarcted hearts

To derive the Sca-1^+^ line, normal hearts (five animals/group) were excised and immediately processed. Isolation was performed by using the Cardiac Stem Cells Isolation kit (Millipore, Billerica, MA, USA), according to Manufacturer's instruction. Following isolation, cells were maintained in cardiac Stem Cell Maintenance Medium (Millipore). For isolation of Sca-1^+^/CD45^+/−^ cells, a flow cytometry-based sorting method was adopted. Briefly, myocardial tissue was digested to obtain a single cell suspension, then labelled with anti Sca-1 and anti CD45 antibodies and finally sorted by using a BD FACSAria II™ Flow-Sorter. Further details about derivation, differentiation and functional characterization of these cells are provided in the online Data S1.

### Histology/Immunofluorescence

Left Ventricle Transversal sections of paraffin-embedded hearts (five animals/group/time-point) were de-waxed and re-hydrated with conventional ethanol series. Gross morphology of the LV wall was revealed by haematoxylin/eosin staining followed by image acquisition under an Axioskop light microscope (Zeiss Italia, Arese, Italy) equipped with a high-resolution digital camera. For IF imaging, de-waxed slides were treated for antigen retrieval, followed by incubation with blocking and primary/secondary antibodies solutions. Three/four fluorescence-stained slides were observed with an LSM710 Confocal Microscope (Zeiss). Further details about histology and IF methods are provided in the online Data S1.

### RNA interference and cell transfection

Validated high-performance purity grade small interfering RNAs (siRNA) against GPR17 were synthesized by Thermo Scientific Dharmacon by using the Acell siRNA design algorithm and a proprietary homology analysis tool. Control siRNA, with a non-silencing oligonucleotide sequence that does not recognize any known homology to mammalian genes, was also generated as a negative control. Cells, at 70–80% confluence, were transfected with siRNA by using Accell delivery medium (Thermo Fisher Scientific, Lafayette, CO, USA). After 24 hrs, the transfection procedure was stopped by cell collection to RNA extraction. Expression of GPR17 and functional analysis were performed as described in the online supplementary material.

### Statistical analysis

All data are expressed as mean ± SEM. Overall comparisons of the treatment groups were performed by using one-way anova method with Newman Keuls post-hoc test. In case of one-to-one comparisons, two tailed unpaired Student's *t*-test was used. *P* values <0.05 value were considered statistically significant and indicated by * in the figures.

## Results

### GPR17 is expressed in stromal Sca1^+^ cells in normal harts, in Sca-1^+^/CD31^−^ cells, and in recruited myeloid cells after MI

**Fig. 1:**
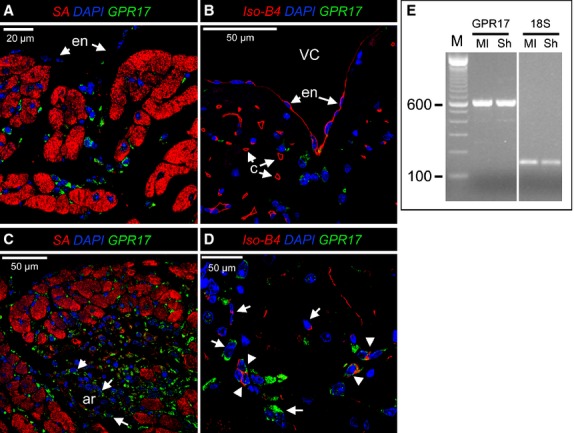
Expression of GPR17 in normal mouse hearts and after infarction. (**A** and **B**) Confocal microscopy images of normal mouse hearts histological sections stained with GPR17, α-Sarc Actin (SA) specific antibodies and TRITC-conjugated Isolectin-B4 (Iso-B4). GPR17 was observed exclusively in interstitial cells between the myocytes and were not associated with capillaries (c). Arrows indicate the endocardial cells (en) covering the ventricular cavity, which were also negative for GPR17 in non-ischaemic mice. (**C** and **D**) the number of GPR17^+^ cells was dramatically increased already at 24 hrs after MI in ischaemic areas. GPR17^+^ cells were also found arterial-like structures (ar, arrows) present in the ischaemic areas. Iso-B4 staining showed that after MI induction, a population expressing GPR17 and labelled with the Lectin appeared in the ischaemic areas (arrowheads). These cells were found in association with groups of cells expressing, alternatively, only one of the markers (arrows). (**E**) RT-PCR analysis of the total RNA extracted from sham (Sh)-operated *versus* infarcted (MI) mice showed no changes in GPR17 before and after MI. The 18s rRNA was used as an internal control.

To localize cells expressing GPR17 in the mouse heart, an IF staining with an antibody directed against the GPR17 C-terminal region was set up. The specificity of this antibody to recognize GPR17 has been previously determined [18]. The GPR17 staining was initially used in combination with the cardiac marker α-Sarcomeric Actin (SA) to discriminate between myocytes and non-myocyte cells. The results (Fig. 1A) identified abundant interstitial cells expressing GPR17; these cells were found in all heart compartments without a confined expression. GPR17 antibody staining was then performed in combination with TRITC-conjugated Isolectin-B4 (Iso-B4), which specifically recognizes endothelial cells and macrophages [19,20] (Fig. 1B). This showed a clearly non-overlapping expression of GPR17 in Iso-B4^+^ cells, thus excluding endothelial or myeloid phenotypes of GPR17^+^ cells in the normoxic heart.

To assess the expression of GPR17 in the ischaemic myocardium, a mouse model of MI by permanent ligation of the LAD coronary artery was used [17]. As shown in Figure S1, in this model, a burst of infiltrating cells appeared in the ischaemic areas as early as at 24 after ischaemia. At this time-point, the presence of GPR17^+^ cells was markedly increased, especially at the infarct border zone (Fig. 1C). Following infarction, a portion of GPR17^+^ cells also displayed reactivity with Iso-B4 (Fig. 1D), suggesting a macrophage phenotype. Finally, the expression of GPR17 was confirmed by RT-PCR in ischaemic *versus* normal hearts (Fig. 1E).

**Fig. 2:**
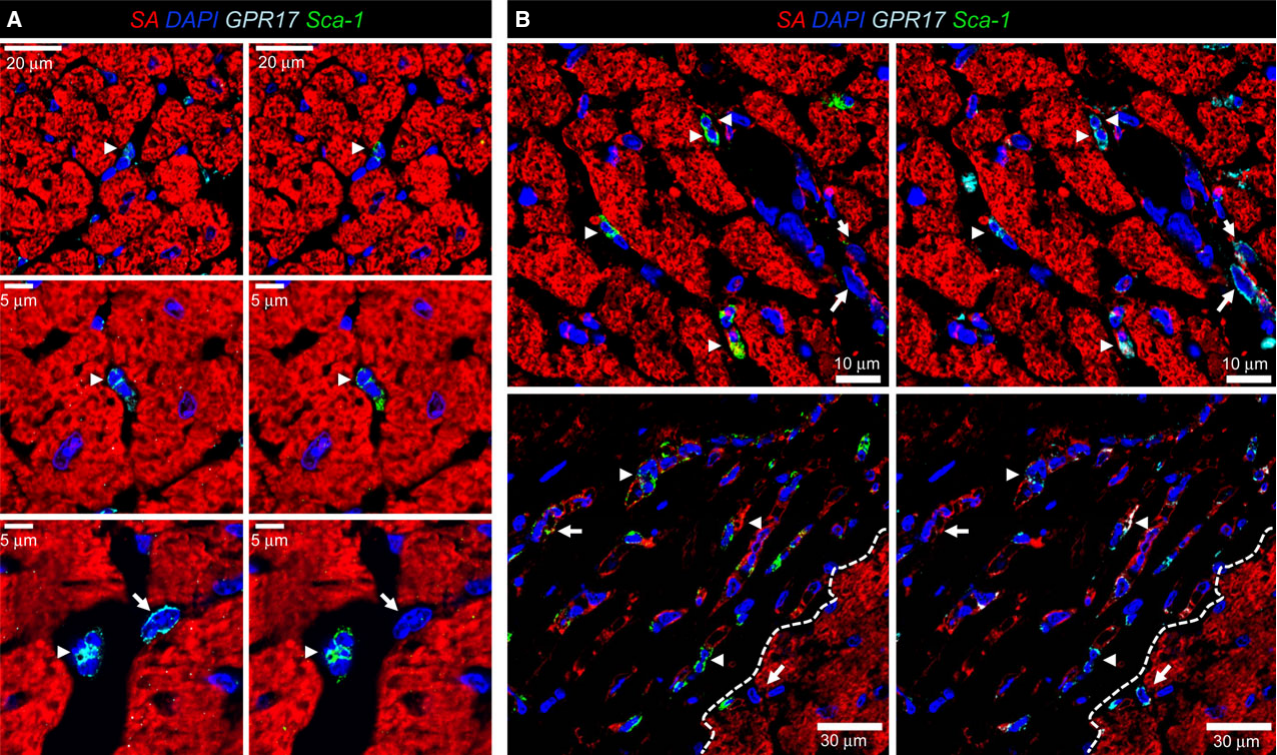
GPR17^+^ expression in Sca-1^+^ cells before and after MI. (**A**) Staining with SA, Sca-1 and GPR17 antibodies showed that GPR17^+^ interstitial cells in the normal heart express the Sca-1 marker (arrowheads). Only occasionally, cells expressing GPR17 were Sca-1^−^. (**B**) The number of GPR17^+^/Sca-1^+^ cells (arrowheads) in the border zone of the infarct and in the ischaemic areas were elevated at 24 hrs after MI. Interestingly, the cells expressing, alternatively, only one of the markers were also increased (arrowheads), suggesting a heterogeneous origin. Dashed line indicates the boundary between the ischaemic and non-ischaemic zone.

The presence of Adenosine receptor A_2B_ has been recently reported in Sca-1^+^/CD31^−^ cardiac cells, implying purinergic signalling in the regulation of these cells [16]. We therefore hypothesized that GPR17 may be expressed, before and after ischaemia, in stromal cells with mesenchymal characteristics [21]. To this aim, an IF analysis was performed to assess the expression of Sca-1 marker in GPR17^+^ cells. The results showed that before MI induction the vast majority of GPR17^+^ cells expressed Sca-1 (Fig. 2A). Because of their paucity, cells expressing Sca-1 in the non-ischaemic ventricular tissue could not be quantified. At 24 and 48 hrs after ischaemia, there was a dramatic increase in the number of cells expressing both markers (Fig. 2B, Fig. S2) and the appearance of GPR17^+^ cells, which did not express Sca-1. Irrespectively of Sca-1 expression, GPR17^+^ cells appeared located in groups apparently migrating just beneath the living myocardial cells. Membrane expression of GPR17 was confirmed by Z-stack reconstruction of confocal images (Fig. S3).

**Fig. 3:**
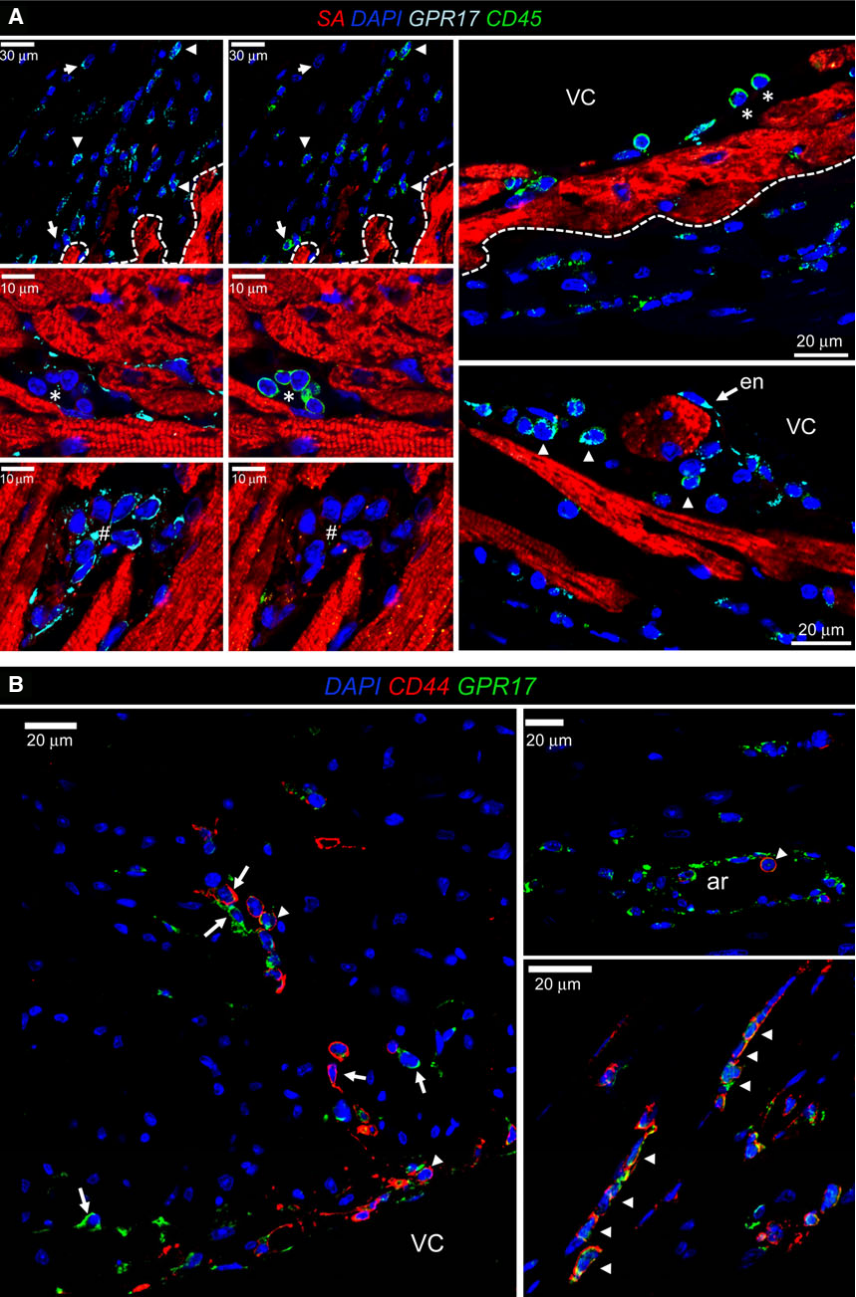
Expression of CD45 and CD44 in subgroups of GPR17^+^ cells. (**A**) Panels on the top left show low magnification of CD45 marker expression in GPR17^+^ (arrowheads) and GPR17^−^ (arrows) cells. The four panels on the bottom left show that some cellular clusters had a mutually exclusive expression of the two markers (* for CD45^+^ and # for GPR17^+^ cells). The high magnification panels on the right show the presence of CD45^+^ cells homing events. These cells (*) adhered to the endocardium (en, arrows) covering ventricular cavity (VC); note that endocardial cells in ischaemic mice expressed the receptor. CD45^+^ cells expressed GPR17 only after invading the sub-endocardial tissue (arrowheads). The dashed line indicates the boundary between the ischaemic and non-ischaemic zone. (**B**) CD44^+^ cells infiltrating the myocardium were observed at 48 hrs post-MI both in the sub-endothelium and in the ischaemic myocardium. Cells expressing both markers (arrowheads) or, alternatively, one of the markers were observed. As shown in the upper right panel, round cells co-expressing GPR17 and CD44 were found associated with the endothelium of an arterial-like vessel (ar) in the ischaemic myocardium. Note the presence of endothelial cells expressing GPR17. VC; ventricular cavity.

The appearance in the ischaemic myocardium of GPR17^+^/Iso-B4^+^ (Fig. 1D) and GPR17^+^/Sca-1^−^ (Fig. 2B and Fig. S2) cells suggested that the receptor characterizes also a circulating inflammatory cells population recruited at short time after infarction. To substantiate this finding, we performed a CD45/GPR17 staining. This indicated the presence of CD45 in groups of GPR17^+^ cells (Fig. 3A), particularly in those located below the endocardium layer (Fig. 3A). To quantify the presence of cells expressing GPR17 in conjunction with the other considered markers, a cell counting on tissue sections was finally performed (Fig. S4). This revealed the most abundant cells to be the GPR17^+^ followed by those Iso-B4^+^. By contrast, the Sca-1^+^ cells were the less abundant.

**Fig. 4:**
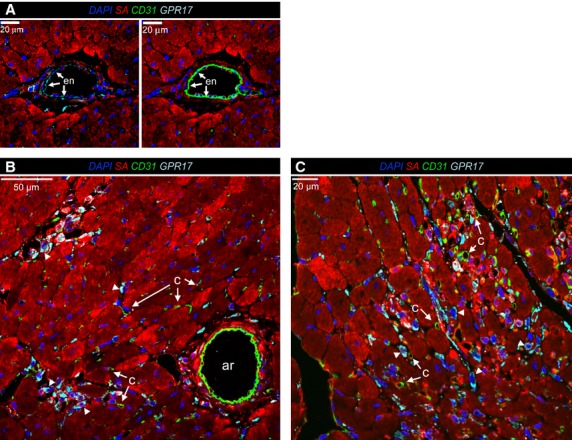
Absence of endothelial marker CD31 in GPR17^+^ cells invading the ischaemic myocardium. (**A**) a GPR17 immunoreactivity was observed in the endothelium (en, arrows) of arterial-like vessels present in the ischaemic border zone. (**B** and **C**) high-power views of the ischaemia border zone, showing that GPR17^+^ cells (arrowheads) did not show CD31 immunoreactivity. This excludes the endothelial phenotype of GPR17^+^ cells invading the myocardium after MI. c: capillaries; ar: arterial-like vessels.

To exclude that GPR17^+^ cells are endothelial cells recruited by ischaemia in the infarcted myocardium, a CD31 immunostaining was performed (Fig. 4). This showed the absence of GPR17 and CD31 co-expression in the infiltrating cells at the border zone of the ischaemic myocardium, although an immunoreactivity for the receptor was clearly found in the endothelium of arterial-like vessels (Figs 3B and 4). Taken together, these results suggest that distinct GPR17^+^/CD31^−^ cellular populations, one deriving from cardiac stromal cells (neither expressing CD45 nor stained with Iso-B4), and another from blood-derived inflammatory cells (expressing CD45 and stained with Iso-B4), are concomitantly recruited in the ischaemic myocardium.

### Cardiac GPR17^+^/Sca-1^+^/CD31^−^/CD45^−^ cells represent a myo-fibroblast (MF) progenitor cell population *in vivo* and *in vitro*

**Fig. 5:**
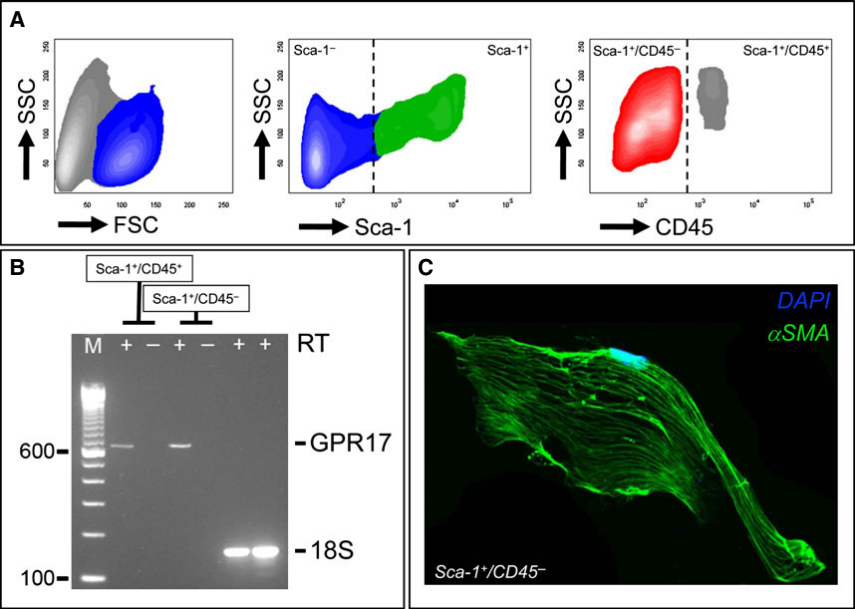
Expression of GPR17 characterizes a MF progenitor cell population in the infarcted heart. (**A**) Flow cytometry plots showing the logical gating strategy adopted to identify and separate Sca-1^+^/CD45^−^ from Sca-1^+^/CD45^+^ in myocyte-free mononuclear cell suspension obtained by enzymatic dissociation of infarcted hearts at 48 hrs post-MI. (B) Sca-1^+^/CD45^+^ and Sca-1^+^/CD45^−^ cells were sorted and analysed by RT-PCR for the expression of GPR17. (**C**) Flow-sorted Sca-1^+^/CD45^−^ cells underwent spontaneous differentiation into MF-like cells, as revealed by polymerization of αSMA fibres.

To determine whether cells expressing GPR17 have cardiac stromal cells characteristics with the potential to differentiate into MF-like cells [22], co-staining with CD44 and Collagen-I was performed (Fig. 3B and Fig. S5). CD44 was present in a high proportion of GPR17^+^ cells, while only a non-quantifiable amount of these cells was found to co-express Collagen-I, suggesting an immature phenotype. This was confirmed by a time-course of MF-specific marker α-SMA (Fig. S6), which started being expressed in the scar only 1 week after infarction [23]. A flow-sorting strategy was then used to separate Sca-1^+^/CD45^+^ from Sca-1^+^/CD45^−^ cells in the ischaemic heart (Fig. 5A). Flow-sorted cells were used for RNA extraction, or to assess differentiation in culture. Results indicated GPR17 expression in both sorted cellular populations (Fig. 5B). In addition, Sca-1^+^/CD45^−^ cells spontaneously differentiated into MFs, as demonstrated by the presence of polymerized smooth muscle actin fibres (Fig. 5C), a hallmark of MFs [24]. As the number and the viability of sorted cells from the ischaemic hearts were not appropriate to perform functional studies, a cardiac Sca-1^+^/CD31^−^ cell line was derived by clonal amplification as already described [21]. Figures S7 and S8, show the principal characteristics of these cells. Consistent with previous characterizations, this cell line expressed high levels of mesenchymal markers CD29, CD44 and CD105 [21], and displayed a relatively high percentage of cells extruding the nuclear marker Hoechst 33342 in a ‘side-population’ (SP) flow cytometric assay [25]. Sca-1^+^ cells did not express haematopoietic lineage markers and the tyrosine kinase receptor c-kit (CD117), the marker characterizing cardiac stem cells and other relevant myocardial-resident cells such as telocytes and stromal cells in mice [26–31] and humans [32–34] (data not shown). The expression of GPR17 in these cells was confirmed by IF, flow cytometry and RNA analyses.

**Fig. 6:**
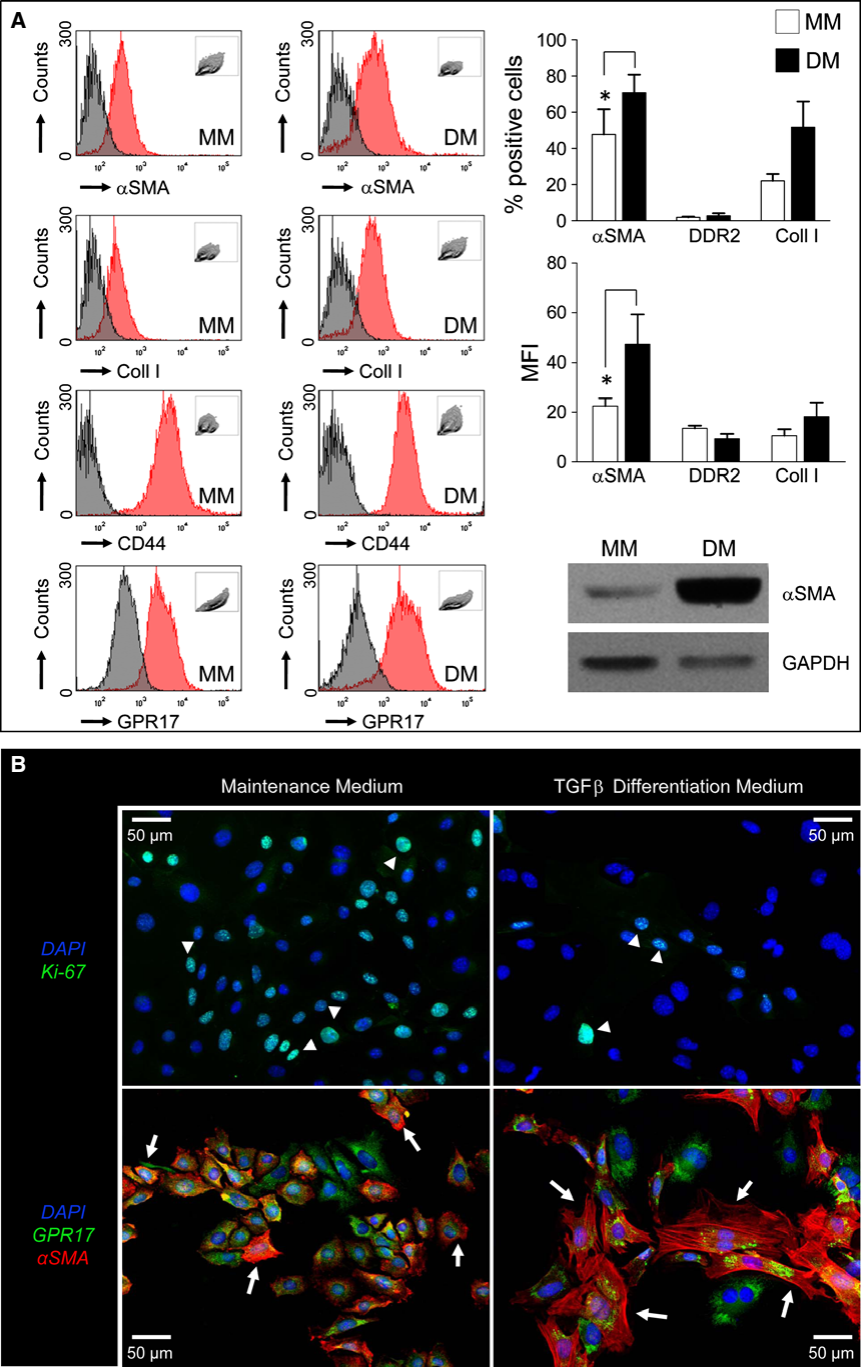
Cardiac GPR17^+^/Sca-1^+^/CD31^−^/CD45^−^ cells represent a myo-fibroblast (MF) progenitor cell population. (**A**) the GPR17^+^/Sca-1^+^/CD31^−^/CD45^−^ cell line obtained from normal hearts (Fig. S6 and S7) was treated with a differentiation medium (DM) containing TGF-β to induce MF differentiation. The treatment caused up-regulation of MF marker αSMA and, although not significantly, Collagen-I. The increase in αSMA expression was also confirmed by the analysis of the mean fluorescence intensity (MFI), as well as by western blotting. DDR2 expression remained low, while CD44 did not change. (**B**) Immunofluorescence with a Ki-67 antibody (upper panels) and αSMA and GPR17 antibodies (lower panels) in maintenance (MM) and differentiation (DM) media revealed that the stromal cell line acquired MF characteristics as characterized by formation of αSMA fibres. Differentiation occurred in concert with a reduction in KI-67^+^ cell number (arrowheads). By contrast, GPR17 was not down-regulated.

The function of the derived GPR17^+^/Sca-1^+^/CD31^−^/CD45^−^ cells as MF progenitors was assessed in differentiation assays by using a medium containing transforming growth factor β (TGF-β), a cytokine playing a pivotal role in activating MFs in the ischaemic heart [4]. Under MF differentiation conditions (Fig. 6), a substantial change in cellular morphology was observed, with an increase in cell size, the adoption of a fibroblast morphology and the development of smooth muscle actin containing stress fibres. Interestingly, treatment with TGF-β also potently inhibited cell proliferation, shown by a decrease in the amount of cells expressing the cell-cycle marker Ki67. Flow cytometry analysis showed that CD44 and GPR17 expression were maintained at high levels in TGF-β-treated cells, while αSMA was significantly up-regulated. Finally, the percentage of Collagen-I^+^ cells showed a clear trend to increase, although it did not reach statistical significance.

### GPR17 agonists promote Sca-1^+^ cells migration *in vitro*

**Fig. 7:**
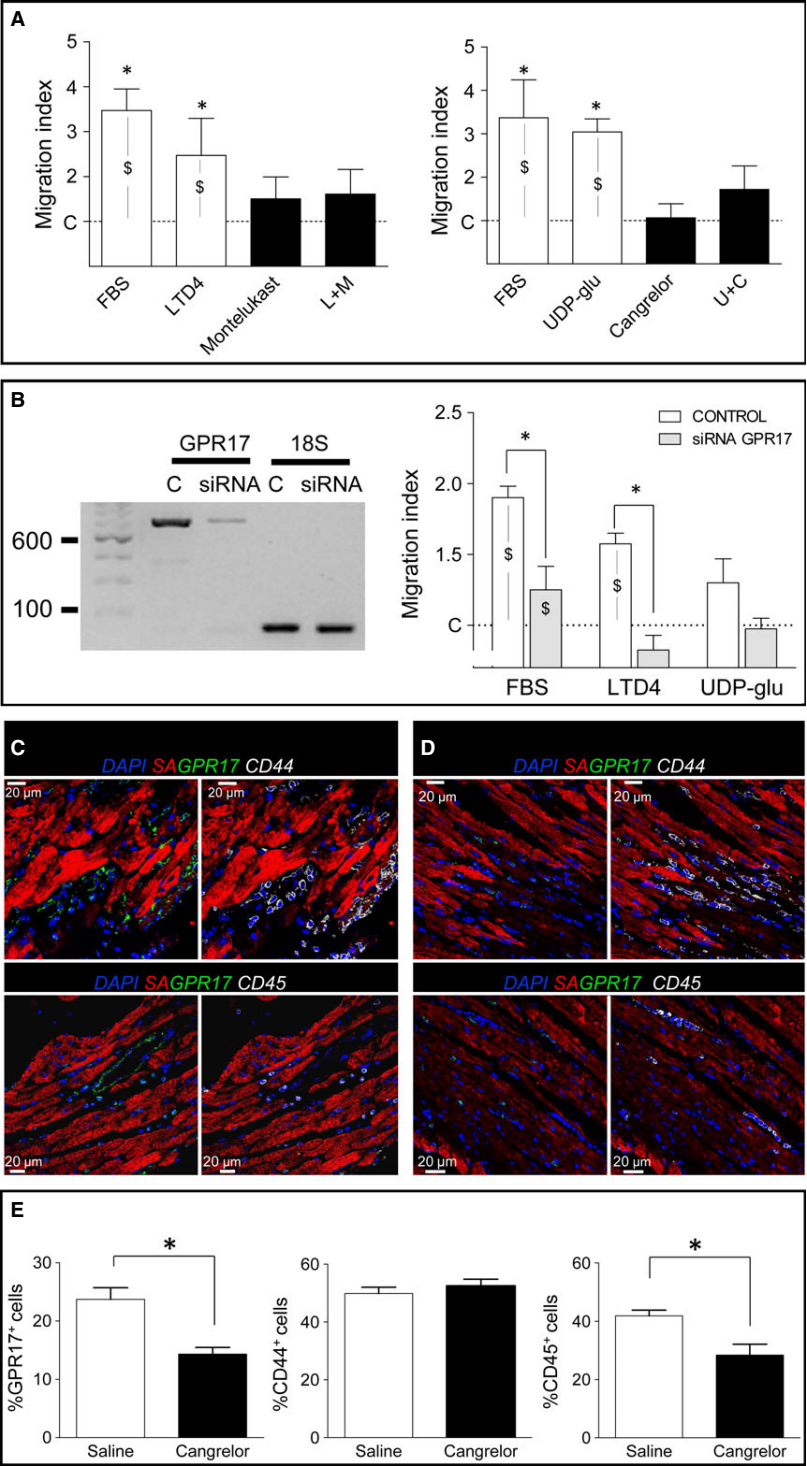
GPR17 promotes migration of Sca-1^+^ cells *in vitro* and *in vivo*. (**A**) A transwell-based migration assay was used to assess the ability of GPR17 agonists LTD4 [L] and UDP-Glucose [U] (purity ≥97% for LTD4 and ≥98% for UDP-Gluc) to induce Sca-1^+^ cells migration. FBS was used as a positive control, while treatments with Montelukast [M] and Cangrelor [C] were used to revert the agonists effect on migration. * indicates significant differences (*P* < 0.05) in the migration level of Sca-1^+^ cells in the presence of FBS, LTD4 and UDP-Glucose *versus* cells treated with the GPR17 antagonists Montelukast and Cangrelor, or the combined agonist+antagonist treatment (L+M; U+C); data were compared by one-way anova with Newman-Keuls post-hoc analysis (*n* > 4). $ indicates significant differences in the migration level of Sca-1^+^ cells in the presence of FBS and GPR17 agonists (LTD4 and UDP-Glucose *versus* cells maintained in serum-free medium during migration (negative control). (**B**) Reduction of Sca-1^+^ cells migration in the presence of small interfering RNAs (siRNA) against GPR17. The RT-PCR on the left shows a reduction in GPR17 expression at 48 hrs after siRNA transfection. Graph on the right indicates the migration index of siRNA treated cells compared to controls. As shown, GPR17 down-regulation had an overall effect on Sca-1^+^ cells motility. On the other hand, receptor down-regulation abolished the migratory activity elicited by LTD4 treatment and reduced, although not significantly, the migration induced by UDP-Glucose. $ indicates a *P* < 0.05 significance level by paired *t*-test comparisons of migration observed in Control siRNA and GPR17-siRNA cells in the indicated conditions (*n* = 4). * indicates a *P* < 0.05 significance level by comparing migration observed in Control siRNA *versus* GPR17-siRNA in the indicated conditions using unpaired *t*-test (*n* = 4). (**C** and **D**) Immunofluorescence detection of GPR17, CD44 and CD45 expressing cells in sections of infarcted mice treated with saline (**C**) or Cangrelor (**D**). (**E**) *In vivo* recruitment of GPR17^+^ and CD45^+^, but not of CD44^+^ cells *in vivo* was reduced by Cangrelor treatment. Data in the bar graph are expressed as percentages of marker^+^ cells/total cell number. Images were acquired and analysed by a double-blind analysis procedure. * indicates *P* < 0.05 by unpaired *t*-test; *n* > 20 independent images on saline-injected (*n* = 3) or Cangrelor-injected (*n* = 3) infarcted hearts stained with GPR17/CD44 or GPR17/CD45 antibody mixture.

The previous results indicated that the clonal cell line could be used *bona fide* as a model to investigate GPR17 functions in cardiac stromal cells. Experiments in the presence of agonists such as UDP-Glucose (100 μM) and LTD4 (100 nM), known to activate the receptor, were then performed. While neither UDP-Glucose nor LTD4 caused significant changes on (*i*) cell cycle, (*ii*) hypoxia-induced apoptosis (data not shown) or (*iii*) TGF-β-induced cells differentiation, treatment with both agonists had a potent effect in migration assays (Fig. 7A). Interestingly, the migratory effect was inhibited by adding two GPR17 pharmacological antagonists, Cangrelor (10 μM) and Montelukast (1 μM). The direct role of GPR17 in Sca-1^+^ cells migratory activity was univocally confirmed by siRNA studies, which showed a reduction in the agonist-dependent cell motility in GPR17 knockout cells (Fig. 7B).

### Reduced recruitment of GPR17^+^ and CD45^+^, but not of CD44^+^, cells in infarcted hearts treated with P2Y antagonist Cangrelor

Given the involvement of GPR17 in Sca-1^+^ cells motility *in vitro*, we tested whether receptor blockade by antagonists alters the early recruitment of cells expressing GPR17, MSC (CD44) or myeloid cells (CD45) markers *in vivo*. The experiment was performed by pre-treating mice with Cangrelor followed by direct drug injection into the infarct border zone. Mice were killed 24 hrs later and the hearts were processed for immunohistochemical detection of the selected markers (Fig. 7C and D). Cell counting on serial confocal microscope images of the ischaemic zone showed a reduced presence of infiltrating GPR17^+^ and CD45^+^ cells in drug-treated *versus* saline-injected mice. By contrast, the drug did not modify the presence of CD44^+^ cells (Fig. 7E).

## Discussion

[9,11][3][35]The importance of Cys-LTs and purinergic signalling in MI is well established. Indeed, studies in the early '90s showed that blockade of leukotriene synthesis or antagonism of Cys-LT receptor alleviates the consequences of MI [9,11]. These data, supported by other findings using anti-inflammatory agents in animal MI models, have led to the hypothesis, never confirmed in clinical trials, that modulation of early inflammatory events might be a feasible way to control the extent of hypoxic ischemic damage (discussed in [3]). P2 receptors have been also proposed as potential therapeutic targets in the cardiac ischemia setting; in particular, the P2X-type receptors with their pleiotropic functions on cardiac cells ion permeability, or the P2Y-type receptors, which are coupled to G_q_, G_s_ or G_i_ proteins and elicit activator or inhibitory actions on PLC or adenylate cyclase in a variety of cardiac cells [35].

### GPR17: a receptor preferentially expressed in resident stromal cells before ischaemia, but shared in inflammatory cells and cardiac mesenchymal cells with MF progenitor characteristics after infarction

GPR17 is a P2Y-like receptor responding to either uracil-nucleotides and Cys-LTs whose presence characterizes various organs susceptible to ischaemic damage (*e.g*. brain, kidney and heart) [12]. Its peculiar pharmacological profile, initially unveiled in heterologous expressing systems, has been independently confirmed by some [36,37], but not other Authors [38]. These contrasting data may be as a result of dependence of the activity tests on the conditions adopted in recombinant ‘artificial’ systems, which potentially give rise to artefacts, especially in the case of constitutive GPR17 activation [39]. On the other hand, GPR17 responses have been more recently fully confirmed by us [18,40,41] and other authors [42] in cells that natively express the receptors, like oligodendroglia [15,18,42] and PC12 cells [40]. GPR17 has been also reported to act as a negative regulator of the Cys-LT1 receptor [36,38]. This suggests that, in addition to responding to cys-LTs, depending on specific pathophysiological conditions, GPR17 can interact with other closely related receptors.

GPR17 functions have been mostly addressed in the brain and the central nervous system, particularly in brain ischaemia or spinal cord injury models [13,43–45]. Interestingly, it was found that *in vivo* modulation of GPR17 by pharmacological or genetic targeting improves the outcome of these injuries, suggesting a central role of the receptor in nervous tissue damage sensing, healing and repair [13,45]. By contrast, except for studies addressing the expression at transcriptional level [12], no further indication has been provided concerning the identity of cells expressing the receptor in the normal heart and, most importantly, whether GPR17 is modulated following induction of MI.

The first part of the present study was therefore designed to address this crucial point. We found that before MI induction, the most abundant cells expressing GPR17 were located in the interstitium between adjacent cardiac myocytes. These cells were distributed in the ventricular myocardium without a preferential location and were not grouped into cellular clusters resembling the typical cardiac stem cell ‘niches’ [46,47]. As the majority of cells expressing GPR17 in non-injured hearts displayed Sca-1 but not Iso-B4 Lectin staining (Figs 1A, B and 2A), we conclude that in normal conditions, the receptor characterizes a unique population of cardiac-resident stromal cells that does not display endothelial or myeloid characteristics. This establishes a striking similarity between the heart and the central nervous system, where GPR17^+^ cells may have a role of damage ‘sensor’ able to activate healing programmes [43].

After the induction of ischaemia, GPR17 was found in at least two distinct populations. The first is represented by Iso-B4^+^ or CD45^+^ myeloid cells, probably recruited from peripheral circulation to the ischaemic tissue (Figs 1C, D and 3A); the other, characterized by Sca-1 and negative for CD31, probably deriving from an intra-cardiac stromal cells store (Fig. 4). This hypothesis is supported by the previous observations that myocardial infarction determines an increase in the amount of Sca-1^+^/CD31^−^ cells [48], and recruitment of a multipotent cardiac MSC population which contributes to cardiac fibrosis [22]. Particularly interesting appeared, in this respect, also the expression of CD44 in GPR17^+^ cells in the infarct tissue (Fig. 3B) and the lack of mature MF marker αSMA at early times after MI, suggesting an immature phenotype. In fact, expression of CD44, in the presence or the absence of CD45, was the principal criterion to discriminate between inflammatory cells homing in the infarcted myocardium from peripheral circulation and stromal progenitors recruited from heart-resident pools [22]. For technical reasons (need of performing indirect GPR17 staining of living cells in conjunction with multicolour membrane marker analysis), it was not possible to proceed with a direct flow-sorting experiment of GPR17^+^/Sca-1^+^/CD44^+^/CD31^−^/CD45^−^ cells from infarcted hearts to resolve the cardiac MSC phenotype and the MF differentiation potency of the GPR17^+^ expressing cells in the infarct. On the other hand, the finding that Sca-1^+^/CD45^−^ cells sorted from infarcted mice (Fig. 5) and that GPR17^+^/Sca-1^+^/CD31^−^/CD45^−^ cells cloned from the normal hearts (Fig. 6) expressed high levels of CD44 and were induced by TGF-β treatment to differentiate into cells with enhanced expression and intracellular polymerization of αSMA protein, strongly suggest the identity of the observed GPR17^+^/CD44^+^ cells in the ischaemic myocardium (Fig. 3B) as heart-resident MSCs, endowed with MF differentiation potency. This latter conclusion is also supported by the absence of c-kit, a marker typical of cardiac-resident stem cells in mice and humans [26,32] and of other stromal cells such as telocytes [28,30].

### Possible function of GPR17 in cardiac-resident MF progenitors

Cys-LTs and purines are well-established initiators of the inflammatory responses. Their role is that of a ‘find-me signal’ attracting the phagocytes to sites of apoptotic cells clearance [49] and inducing a chemokine ‘milieu’ necessary for sequential chemo-attractant cascades involved in *pro*- inflammatory phases associated with tissue damage, innate host-defence, and autoimmunity [50]. Prompted by our preliminary investigations showing that blockade of Cys-LTs signalling alleviates the extent of hypoxic stress-related cardiac fibrosis [51], and other reports suggesting the relevance of Adenosine signalling in the protective function of cardiac stromal cells [16], we determined the function of GPR17 in the cloned cardiac Sca-1^+^/CD31^−^ line. While the treatment of the cells with known Cys-LT/purinergic agonists (LTD4 and UDP-gluc) *in vitro* did not have an effect on proliferation and hypoxia-induced apoptosis (data not shown), both ligands exerted a potent chemotactic effect *via* GPR17 activation. This was shown by reversion of the migratory effect by co-treatment with known GPR17 pharmacological antagonists such as Montelukast and Cangrelor, as well as by a more specific siRNA knockout approach (Fig. 7). In our experiments, we also investigated whether treatment with the two ligands determined modifications in the TGF-β-induced MF differentiation programme. However, we did not observe remarkable differences (data not shown). Altogether, these findings point to a specific GPR17 role in chemotactic guidance of stromal cells towards the ischaemic sites.

A preliminary assessment of a possible *in vivo* GPR17 function was provided by treating infarcted mice with systemic and intra-myocardial injection of the GPR17 antagonist Cangrelor, followed by analysis of the principal cell types recruited in the forming scar (Fig. 7). The blockade of purinergic signalling transmission by Cangrelor led to an interesting and unexpected imbalance between the amount of the recruited cells in control *versus* treated mice. In fact, Cangrelor treatment determined a significant reduction in the number of GPR17^+^ cells and myeloid cells, characterized by CD45 expression, but not that of CD44^+^ cells (Fig. 7). As Cangrelor targets the purinergic, but not the Cys-LT GPR17 signal transduction pathway, this suggests the existence of redundant mechanisms, possibly linked to the dual receptor GPR17 nature. Alternatively, an antagonistic function of GPR17 signalling in distinct stromal and myeloid cells may justify the observed imbalance between cardiac-derived *versus* blood-derived infiltrating cells.

In summary, our results suggest a potential role of the Purinergic and Cys-LT GPR17 receptor in the early response of cardiac stromal cells to ischaemia. This is in agreement with a very recent study showing the involvement of the Adenosine A_2B_ receptor in cardioprotection by Sca-1^+^ stromal cells [52]. Whether selective modulation of GPR17 signalling in cardiac-resident stromal cells translates into beneficial treatments to reduce the extent of myocardial fibrosis and to limit the functional consequences of heart ischaemia is still a matter of speculation, and it is the subject of our current analyses.

## Supporting information

**Figure S1**. Left ventricle transversal sections showing the infract region at 24 and 48 hrs after ligation of the coronary artery. **Figure S2**. Evaluation of GPR17^+^ and Sca-1^+^ cell populations at 48 hrs after MI by immunofluorescence analysis of transversal sections (see Fig. S1 for sections orientation) of the ischaemic LV zones. **Figure S3**. Z-stack confocal image of the ischaemic myocardium 24 hrs after ischaemia. **Figure S4**. Counting of cells single positive (A) or double/triple positive (B) cells for the indicated markers, after staining with multicolour immunofluorescence and confocal analysis. **Figure S5**. GPR17^+^/CD44^+^ cells invading the infracted myocardium do not express mature MF marker Collagen-I. **Figure S6**. Immunofluorescence showing expression of GPR17 in conjunction with mature myofibroblasts marker αSMA in the ischaemic myocardium at 48 hrs (48h; upper panels) and 1 week (1W; lower panels) after MI. **Figure S7**. Phenotypic characterization of Sca-1^+^ cell line. **Figure S8**. Stability of the Sca-1^+^ cells line and quantification of mesenchymal marker expression. **Data S1**. Detailed materials and methods.
