# Supplementary material for: Expression of dual Nucleotides/Cysteinyl-Leukotrienes Receptor GPR17 in early trafficking of cardiac stromal cells after myocardial infarction
Source: J Cell Mol Med. 2014 Jun 7;18(9):1785–96. doi: 10.1111/jcmm.12305 (PMC4196654; doi:10.1111/jcmm.12305)
Supplement: Supplementary file 1 [file jcmm0018-1785-SD1.pdf]

Involvement of dual Nucleotides/Cysteinyl-Leukotrienes Receptor GPR17 in early trafficking of cardiac stromal cells after myocardial infarction

SUPPORTING INFORMATION

Detailed Materials and Methods

Procedures for heart harvesting and tissue preparation

Cardiac magnetic resonance was used at 24hrs from surgery to discriminate mice with a significant reduction in ejection fraction (≤ 45%) by using a 4.7 T vertical-bore MR magnet (Bruker Avance II 200) spectrometer with micro imaging accessory. After 24, 48 and 72 hrs from surgery 5 animals per group at each time point were sacrificed. The abdominal aorta was cannulated, the heart was arrested in diastole with a solution of CdCl2 (0.1M) and KCl (1M), retrograde perfused for 10 min with 0.01M phosphate saline buffer (PBS) and then with 4% (vol/vol) phosphate-buffered formalin for 10 min and post-fixed in 4% phosphate-buffered formalin for 24 hours. Fixed hearts were dehydrated through graded ethanol series, embedded in paraffin and sectioned (4 μm thickness).

In vivo pharmacological study

Thirty C57BL/6 female mice were injected twice daily subcutaneously with Cangrelor (10mg/kg provided by The Medicines Company, Parsippany, NJ, USA) or saline. After after 48hrs they were anesthetized and subjected to permanent left anterior descending (LAD) coronary artery ligation. They contextually received two injections of the drug (3μg/animal) or saline solution at the level of the infarct border zone. Twenty-four hrs after surgery, both groups were sacrificed. Fixed hearts were sectioned to be analysed by histology and immunofluorescence (see below).

Derivation of Sca-1$^+$/CD31$^-$ cell line

Immediately after dissection, hearts (n=5) were rinsed into Cardiac Stem Cell Isolation Buffer (Millipore, USA). Isolated myocardial tissue was then finely minced with a scalpel and incubated for 45 minutes at 37°C using Cardiac Tissue Dissociation Buffer (Millipore, USA). After digestion, the cell suspension was filtered using 100μm mesh nylon filters (Millipore, USA) and placed in a density gradient solution. Following centrifugation, the upper clear phase containing the mesenchymal stem cell suspension was collected. The cells were finally seeded into uncoated Petri dishes (Corning, Italy) containing cardiac Stem Cell Maintenance Medium (Millipore, USA). After 48 hrs. the medium was changed to remove dead non-adherent cells. When reaching a 70% confluence, cells were split 1:3 and seeded into 10 cm uncoated Petri dishes (Corning, Italy) until reaching 70% confluence. Sca-1 expression was controlled by cell incubation with anti-Sca1 monoclonal Ab, (BD Pharmingen, Italy) followed by flow cytometry analyses.

Sca-1$^+$ cell line was obtained by repeated passaging of primary cardiac mesenchymal cells. After about 30 days, clones of proliferating cells expressing Sca-1 at high levels were found in the culture dishes and subcultured as described [1,2]. For detection of Sca-1$^+$ progenitor characteristics, cells from the growing clones were detached by incubating for 3 minutes at 37°C in 5% CO_2 atmosphere with cell dissociation buffer (Sigma, Italy) and re-suspended in DMEM supplemented with 10% FBS (Euroclone, USA). Cells were treated with 10 µg/ml Hoechst 33342 with/without 50 µM Verapamil for 90 minutes. Immediately after cells were stained with Isotype antibody or Sca-1 antibody (see before), analyzed with a BD FACSAria IIU ™ cell sorter. Cells were used between passage 4 and 10.

Cell characterization by flow cytometry

Cells were incubated with the following antibodies recognizing cell surface markers: Sca1-FITC (BD Pharmingen, Italy), Sca1-APC (R&D System, USA), CD44-PE (BD Pharmingen, Italy), CD29-PE (R&D System, USA), CD31-PE (BD Pharmingen, Italy), CD105-FITC (R&D System, USA), Ly-6A/E-FITC (BD Pharmingen, Italy). Staining with the relative Isotype antibodies was used as a control. To check the hematopoietic lineage profile of Sca-1 positive cells, cells were incubated with the APC mouse lineage antibody cocktail or the relative isotype control. To analyze intracellular markers, cells were fixed using Cytofix/cytoperm Fixation/Permeabilization Kit (BD, San Jose), incubated with antibodies to Collagen type I (Rockland Immunochemicals, Gilbertsville, PA), DDR2 (Genex Bioscience, Hayward, CA), Ki67 (Abcam, Cambridge, MA), GPR17 (Cayman Chemical, USA), α-SMA (Sigma-Aldrich, St. Louis, MO) or control IgG. All samples were then washed 3 times with PBS (Lonza, Italy), incubated with the appropriate secondary antibodies (Abcam, Cambridge, MA), and again washed 3 times with PBS before cytometer analysis. Cells were analysed with a FACScalibur flow cytometer (BD Pharmingen, Italy), using the Cell-QUEST software.

In vitro Sca-1$^+$ cells differentiation and immunofluorescence analysis

To induce MF differentiation, Sca-1$^+$ cells were seeded into chamber slides (Lab-Tek) cells for 3 days into a MF differentiation medium consisting of DMEM:F12/10% FBS containing 10 ng/mL TGF-β1 (R&D Systems). To induce cardiac differentiation, Sca-1$^+$ cells were cultured for 3 weeks in cardiac differentiation medium as previously described. The differentiation capacity toward different cell lineages was verified by morphology changes and immunostaining. For immunostaining cells were fixed with 4% paraformaldheyde in PBS followed by an overnight incubation at 4 °C into a blocking solution, consisting of PBS containing 5% serum and 0.3% Triton-X100. Thereafter, cells were incubated with primary antibodies for alpha-Smooth Muscle Actin (α-SMA) and phalloidin-TRITC (Sigma-Aldrich). Alexa 488-conjugated secondary antibody (Invitrogen, CA) were used. Nuclear staining was performed by incubating cells with Hoechst 33342 (Sigma-Aldrich, Italy). Cells images were acquired with an Axio Observer Z1 microscope equipped with Apotome image deconvolution system and Axiovision software (Carl Zeiss, Germany).

Migration Assay

To assess migratory ability of Sca-1^+ cells line, cells were first subject to overnight cell starvation. They were thereafter re-suspended at a 0.4 x 10^6 cells/ml concentration in serum-free medium and plated into a 8 µM polycarbonate membrane transwell (Corining) placed in the migration chamber. GPR17 agonists (UDP-Glucose 100µM; LTD4 100nM) in the presence or the absence of the two receptor pharmacological antagonists Cangrelor (10µM) and Montelukast (1µM), or serum (10%) used as a positive control were directly added to the lower well of the 24 well migration plate, while serum-free medium and 10% FBS containing medium were used as negative and positive controls, respectively. Following a 16 hrs incubation period quantification was performed by crystal violet staining followed by 590 nm absorbance measuring into a microplate spectrophotometer system (Mithras LB940, Berthold technologies, Bad Wildbad, Germany).

Cell sorting

Isolated myocardial tissue was cut into 1-2 mm^3 pieces, washed with PBS (Lonza, Italy) and incubated 4 times for 40 minutes at 37°C in a solution containing 1mg/mL Liberase (Roche, Italy). After digestion, the cell mixture was filtered using 70µm mesh nylon filters (BD-biosciences, Italy) and the cells were finally resuspended into PSB containing 0.1% BSA (Gibco, USA) and 2mM EDTA (Gibco, USA) for 15 min in the presence of fluorescein isothiocyanate (FITC)-conjugated monoclonal antibody directed against mouse Sca-1 receptor (BD Pharmingen, Italy), at 10 µg/mL concentration, and a Peridinin Chlorophyll Protein Complex (PerCP)-conjugated monoclonal antibody directed against CD45 antigen (BD Pharmingen, Italy), at 10 µg/mL concentration. Sorting setup and appropriate gating was established each time using cells appropriately labeled with FITC/PerCP-conjugated isotype control antibodies at the same concentration. To minimize cell death and maximize recovery, cells were sorted with a low pressure (20 PSI) protocol and using a 100 µM noozle, using BD FACSAria IIU ™ cell sorter. Data analysis was performed with FACS DiVa Software™ version 6.1.3 (BD San Jose, CA). Sorted $Sca1^+CD45^+$ cells and $Sca1^+CD45^-$ were sub-cultured into chamber slide plates (Corning, Italy) and analyzed for immunofluorescence staining. Alternatively they were sorted directly in Tripure isolation buffer (Roche, Italy) for proceeding witth RNA extraction for GPR17 RT-PCR analysis.

RT-PCR

Total RNA was eluted using 20-30 µl of RNase-free water. 1 µg sample was treated with DNase I (Invitrogen Life Technologies, Milano, Italy) and then reverse transcribed (RT) and amplified using SuperScript® III One-Step RT-PCR System with Platinum®Taq (Invitrogen, CA). RT-PCR analysis was performed using Thermo-cycler PCR System (Bio-Rad, Italy). The RT-PCR-reaction included 0,3-5 ng of template cDNA, 0.25µM of each (forward and reverse) primers, cycling conditions were as follows: 55 °C RT-step for 30 minutes, 94°C enzyme activation for 2 min, followed by 40 cycles of amplification (15''at 94°C, 30'' at 60°C annealing, 90''at 68°C, as denaturation, annealing and extension steps). PCR templates (5 µl) were loaded in Agarose gel (2%) for 1 hour (60V) and were analyzed using Gel DOC 2000 imager (Bio-Rad, Italy). The sequence of the primers used in the amplification protocols are as follows:

GPR17F: 5’-GCTCTTCGCCTGCTTCTACC-3

GPR17R: 5’-GCGGACGGCTTTATTCTTGA-3’

18S Fw: 5’-CGGCTAC- CACATCCAAGGAA-3’

18S Rv: 5’-CCTGTA TTGTTATTTTTCGTCACTACCT-3’

Gel electrophoresis/immunoblotting analysis

Protein extract homogenization was performed in Laemmli buffer (1 mmol/L phenyl-methane-sulfonyl-fluoride, PMSF; 10 µg/mL aprotinin, and 5 mmol/ L benzamidine). Proteins were electrophoretically separated on a 12% SDS-polyacrylamide gel and transferred to nitrocellulose membranes. After blocking with 5% dry milk in TBS buffer (20 mM Tris pH 7.4 ,150 mM NaCl), filters were probed with anti-GPR17 (1:1000) (Cayman Chemical, USA), anti-β Actin (1:1000) (Sigma-Aldrich, St. Louis, MO) and anti- α-SMA(1:200) (Abcam, Cambridge, MA) antibodies prepared in TBS buffer containing 5% milk and 0,3 % tween 20. Appropriate peroxidase-coupled secondary antibodies were applied for 1hr at room temperature and peroxidase signals were detected using a chemiluminescent substrate (Pierce, Rockford, IL). Western analyses were run in triplicate.

Histology/IF tissue sections analysis

Heart sections, de-waxed/rehydrated, were incubated for 45 mins at room temperature in 10% normal goat serum (Dako) in PBS 0.01M (Lonza, Italy) and 0.1% Triton X-100. Heat-induced epitope retrieval was performed (except for SCA-1 detection) in sodium citrate buffer (10mM sodium citrate, 0.05% Tween 20, pH 6.0). Primary antibodies were applied overnight at 4°C and secondary antibodies for 2 h at room temperature. All antibodies were prepared in PBS and 0.1% Triton X-100. For nuclear staining, the sections were incubated with Hoechst 33258 (2.5 g/ml; Invitrogen) in PBS for 15 min, before slide covering and confocal microscopy observation. For co-localization studies polyclonal rabbit anti-GPR17 (1:400) generated as previously described [3] was used in association with a selection of primary antibody: mouse anti alpha-sarcomeric actin (1:800, Sigma Aldrich), rat anti-CD45 (1:10, BD Biosciences), rat anti-CD44 (1:40, Abcam), rat anti- Sca-1 (1:400, R&D Systems), rabbit anti-collagen I (1:6000, Rockland), rabbit anti-CD31 (1:500, Abcam), mouse anti-SMA, (1:400, clone 1A4 Sigma), and Isolectin-B4 HRP conjugated (1:100, Sigma). For fluorescence detection of primary antibody binding, goat anti-rabbit AlexaFluor 555-conjugated secondary antibodies (1:600), goat anti-rabbit AlexaFluor 633 (1:600), goat anti-mouse AlexaFluor 488 (1:400), goat anti-mouse IgM AlexaFluor 633 (1:600), goat anti-rat AlexaFluor 488 (1:400) and goat anti-HRP TRITC (Jackson Immunoresearch) were used (all Invitrogen). When primary antibodies developed in rabbit (collagen I and CD31) were co-stained with anti-GPR17, they were detected with the high sensitivity tyramide signal amplification kit according to manufactory instructions (Perkin Elmer).

Supplementary Figures

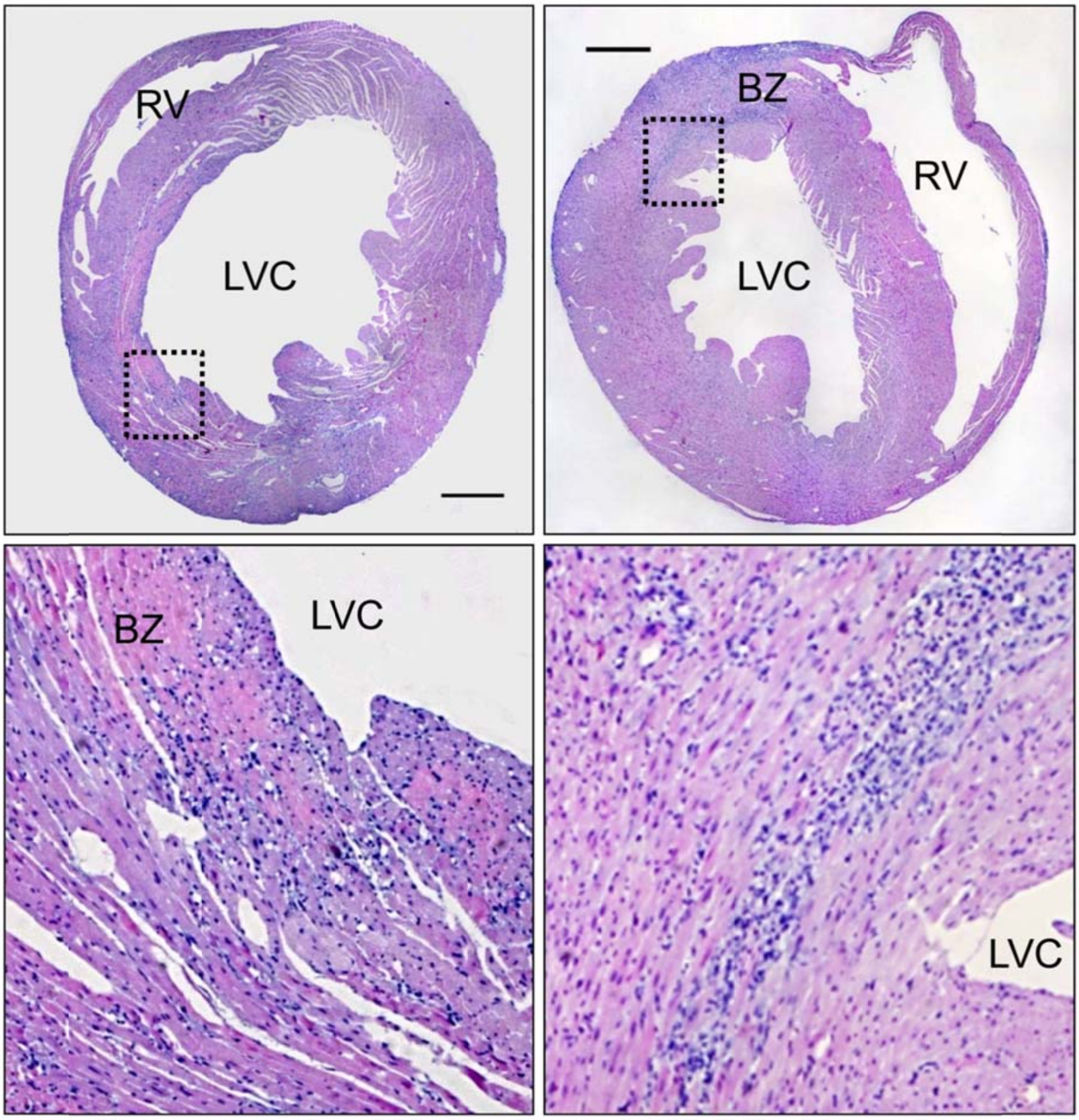

Fig. S1. Left ventricle transversal sections showing the infarct region at 24 and 48 hrs after ligation of the coronary artery. Upper panels show representative images of MI hearts at 24 (left) and 48 hrs (right) after MI transversally cut and stained with Hematoxylin/Eosin staining. The lower panels indicate the regions where myocardial infiltration was observed and where GPR17^{+} cells were found. RV: right ventricle; LVC: left ventricular cavity; BZ: border zone. Scale bar: 1mm.

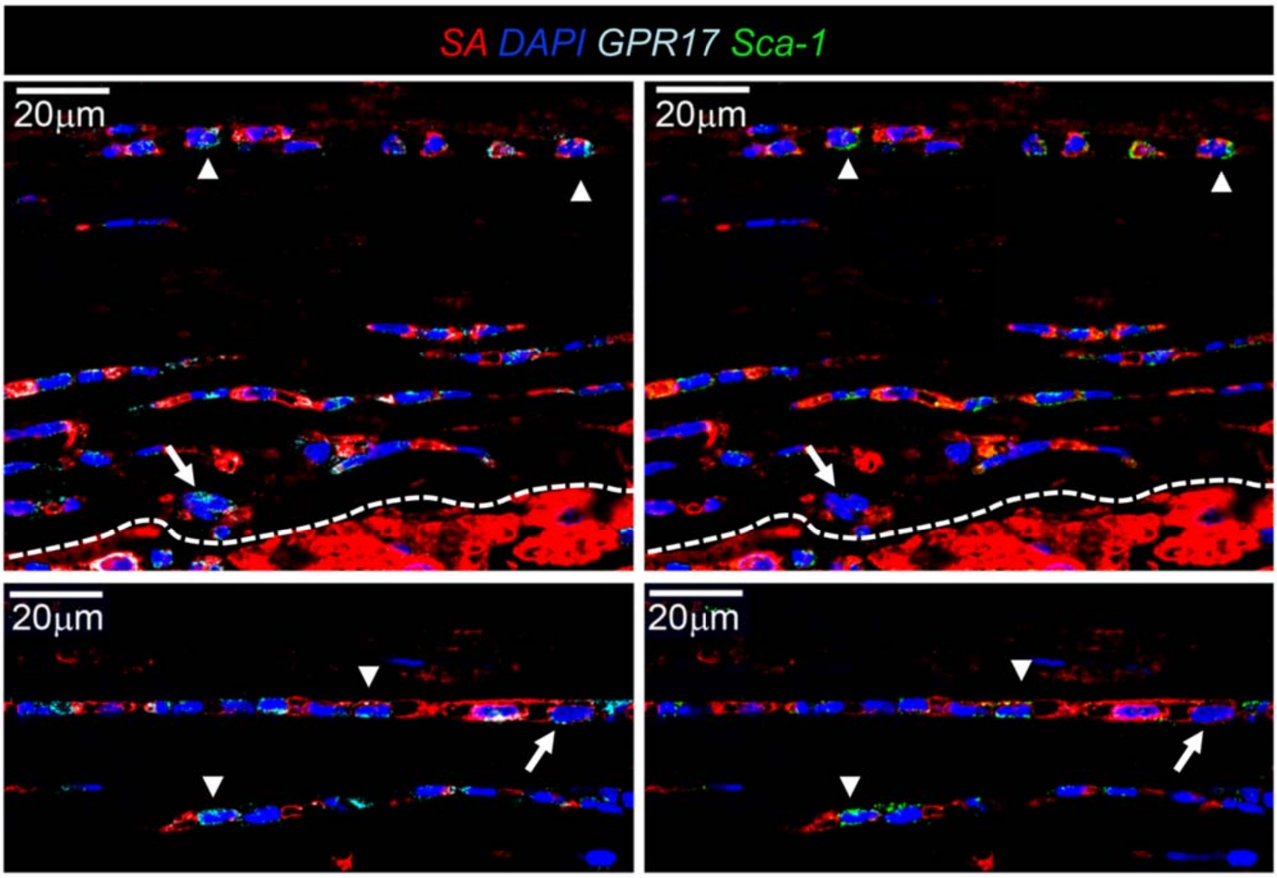

Fig. S2. Evaluation of GPR17^{+} and Sca-1^{+} cell populations at 48 hrs after MI by immunofluorescence analysis of transversal sections (See Fig S1 for sections orientation) of the ischemic LV zones. Cells expressing GPR17 and Sca-1 are indicated by arrowheads. Note the presence of GPR17^{+} cells which do not express Sca-1 (arrows). The bracketed lines indicate the boundary between the ischemic zone and the viable myocardium.

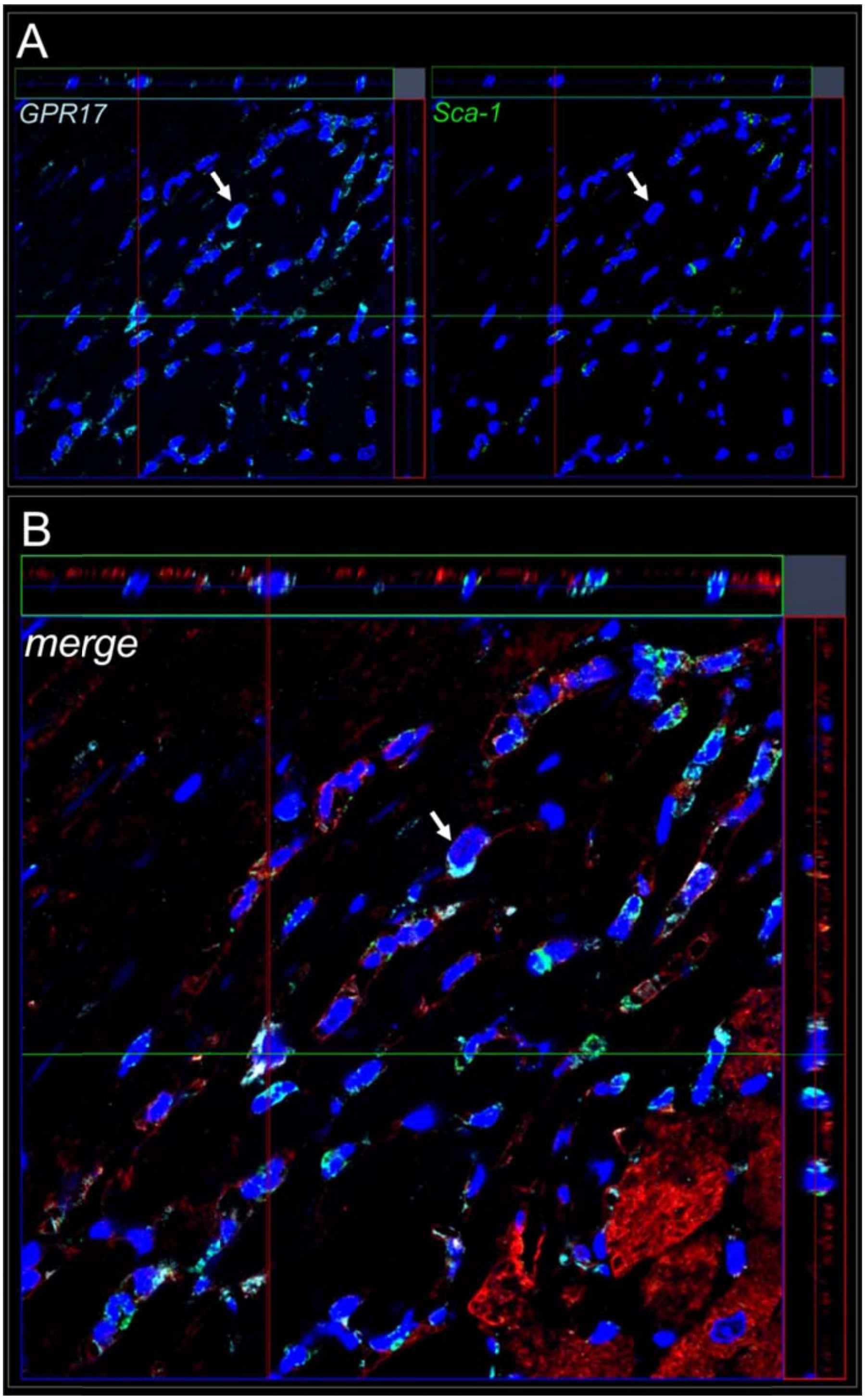

Fig. S3. Z-stack confocal image of the ischemic myocardium 24 hrs after ischemia. (**A**) panel showing separately the GPR17 and Sca-1 signal Z-projection along the X (green) and Y (red) axes. (**B**) merge of the two fluorescent channels, plus α-SA staining (red), showing membrane localization of the two markers in cells invading the infarcted myocardium. Note the presence of one cell clearly expressing GPR17, but not Sca-1 (arrows). GPR17, light blue fluorescence; SA, red fluorescence; Sca-1, green fluorescence; nuclear staining (DAPI), blue fluorescence.

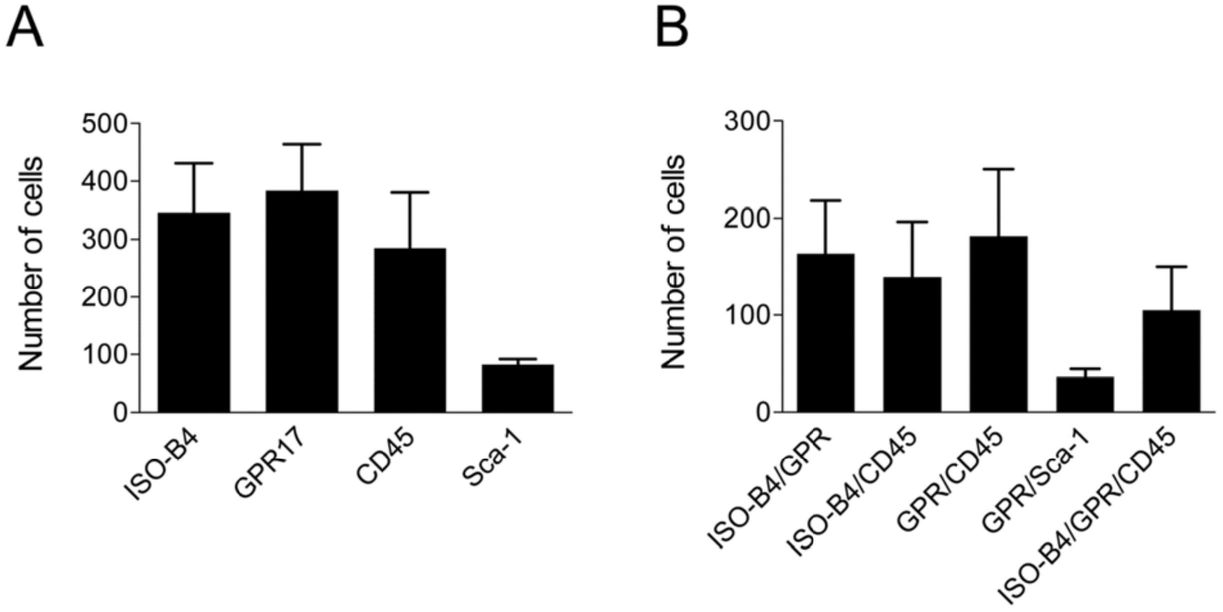

Fig. S4. Counting of cells single positive (**A**) or double/triple positive (**B**) cells for the indicated markers, after staining with multicolor immunofluorescence and confocal analysis. The indicated numbers are the cell counts per fixed area section (~0.7 mm^2) of the scar tissue at 48 hrs *post*-MI; n=3.

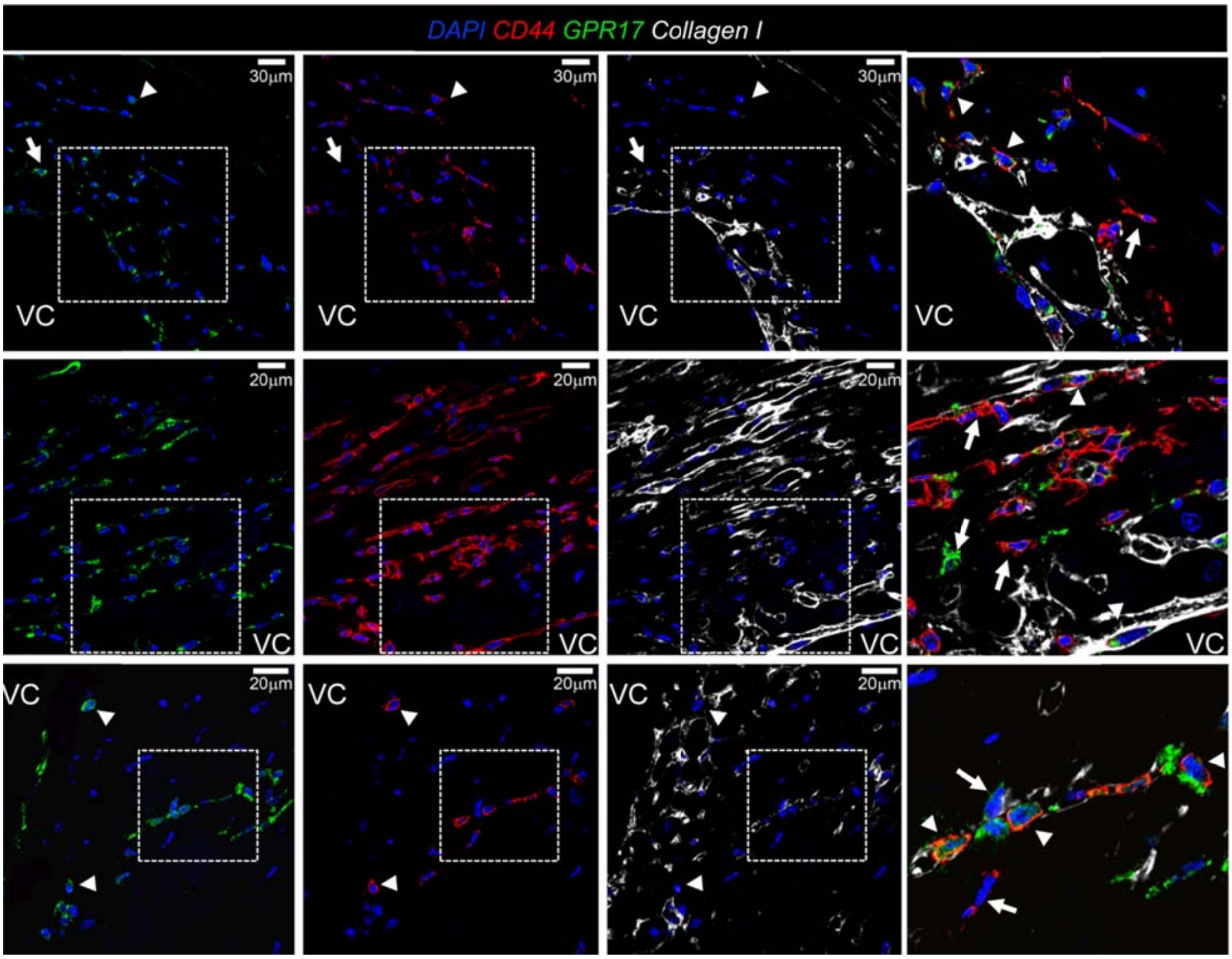

Fig. S5. GPR17^{+}/CD44^{+} cells invading the infarcted myocardium do not express mature MF marker Collagen-I. The image shows three different examples of heavily infiltrated ischemic myocardium zones in sections stained with anti-CD44, -GPR17 and –Collagen-I antibodies. Three series of individual fluorescence are shown for each antigen. The regions indicated in the dashed line boxes are magnified in the right, where arrows indicate cells expressing only one antigen, and arrowheads indicate cells expressing the three markers. Overall, the cells showing a triple fluorescence were a minority.

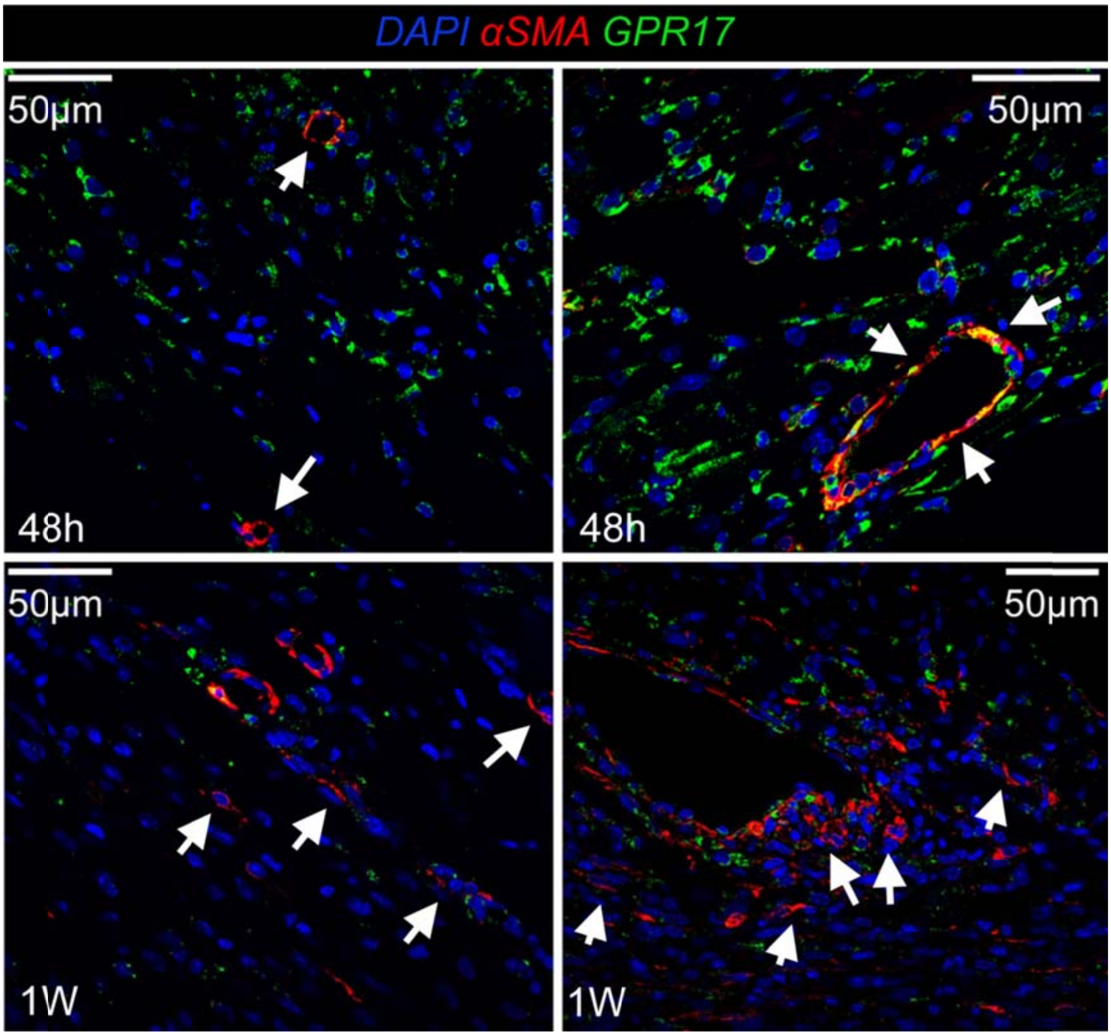

Figure S6. Immunofluorescence showing expression of GPR17 in conjuction with mature myofibroblasts marker αSMA in the ischemic myocardium at 48 hrs (48h, upper panels) and one week (1W, lower panels) after MI. As shown, expression of αSMA was confined to vascular structures at early times after infarction and was not expressed in GPR17^{+} cells (arrows in upper panels). By contrast, cells with mature myofibrobast characteristics were observed starting at one week after MI (arrows in lower panels). This suggests an immature phenotype in the GPR17^{+} cells invading the ischemic myocardium at early stages after coronary artery ligation.

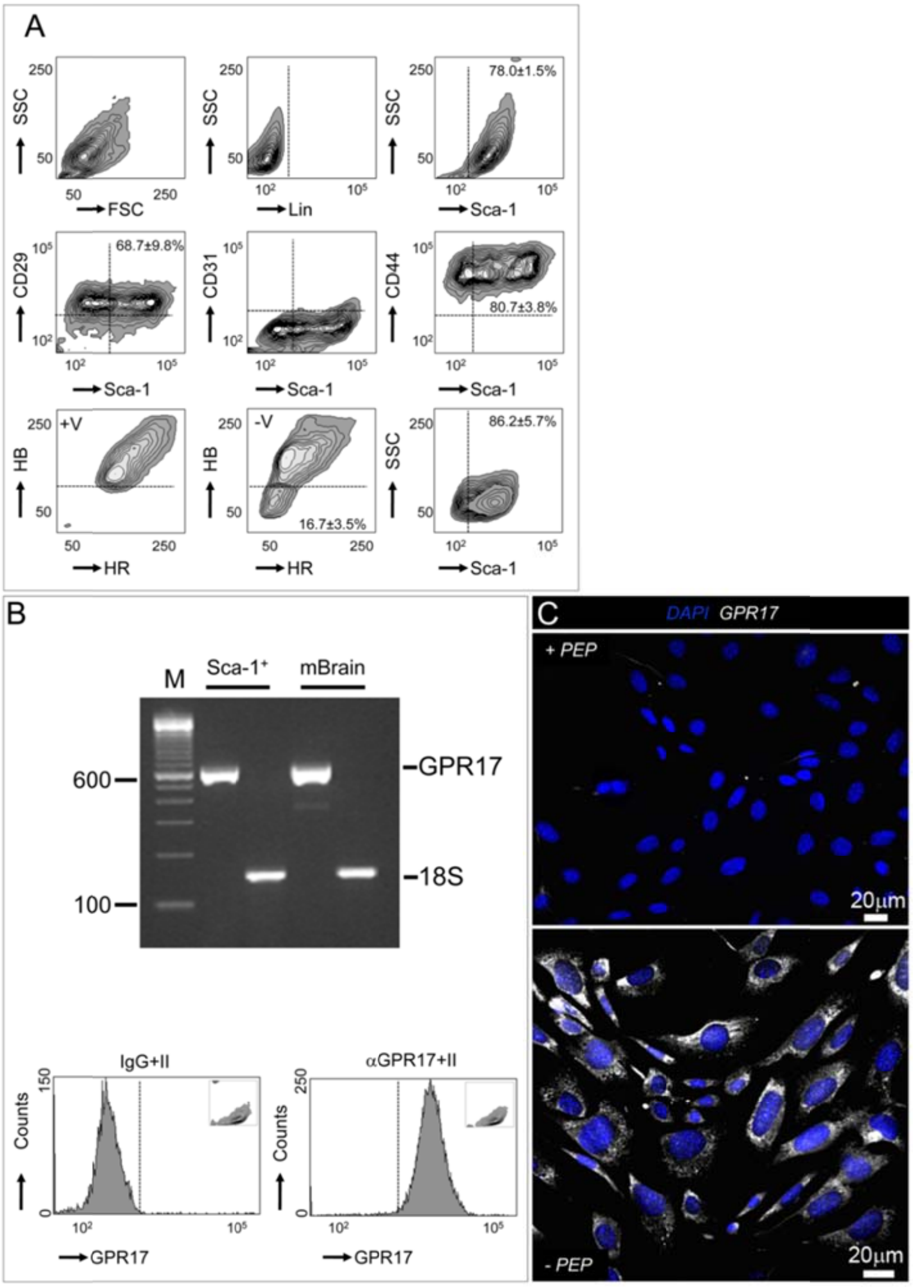

Fig. S7. Phenotypic characterization of Sca-1$^+$ cell line. (**A**) Flow cytometry analysis of the Sca-1$^+$ cell line showing expression of CD29 and CD44 and side population (SP) cells, as detected by Hoechst33342 extrusion , which disappeared with verapamil (V) treatment. Sca-1$^+$ cells did not express EC marker CD31. Plot on the lower right indicates the expression of Sca-1 in SP gated cells. (**B**) RT-PCR detection of GPR17 in Sca-1$^+$ cells. Brain tissue was used as positive control. (C) Sca-1$^+$ cells expressed GPR17 protein, as shown by flow cytometry with an indirect labeling using a FITC-conjugated secondary antibody (II) after staining with unrelated IgGs or the anti-GPR17 antibody. (**C:** Immunofluorescence staining of Sca-1$^+$ cells incubated with anti-GPR17 antibody in the presence (+*PEP*) or the absence(–*PEP*) of an antibody binding inhibiting peptide.

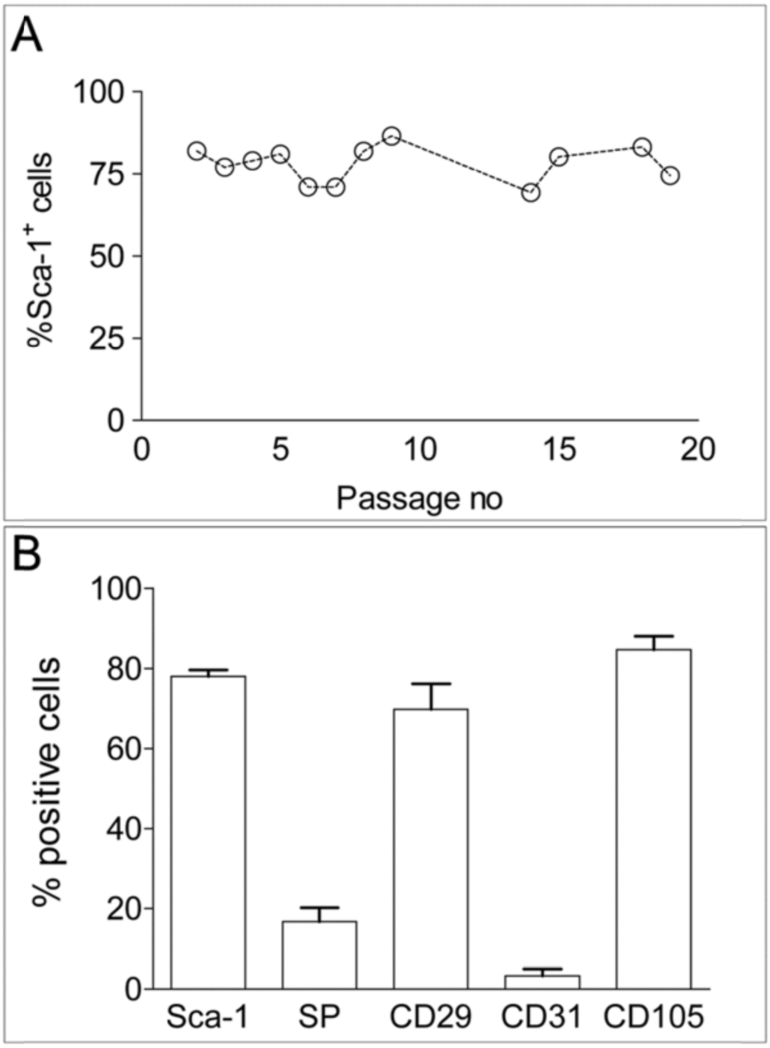

Fig. S8. Stability of the Sca-1$^+$ cells line and quantification of mesenchymal marker expression. Stability of the Sca-1$^+$ cells line and quantification of mesenchymal marker expression. **A:** The expression of the Sca-1 marker was tested at different passage numbers. This showed a slight fluctuation around 75% positivity up to passage 20. **B:** Quantification of CD31 and CD105 markers expression. The bar graph also shows the percentage of Sca-1$^+$ cells and that of cells actively extruding Hoechst33342, recognized as SP cells.

References

1. **Matsuura K, Honda A, Nagai T, Fukushima N, Iwanaga K, Tokunaga M, Shimizu T, Okano T, Kasanuki H, Hagiwara N, Komuro I.** Transplantation of cardiac progenitor cells ameliorates cardiac dysfunction after myocardial infarction in mice. *J Clin Invest*. 2009; 119: 2204-17.
2. **Matsuura K, Nagai T, Nishigaki N, Oyama T, Nishi J, Wada H, Sano M, Toko H, Akazawa H, Sato T, Nakaya H, Kasanuki H, Komuro I.** Adult cardiac Sca-1-positive cells differentiate into beating cardiomyocytes. *J Biol Chem*. 2004; 279: 11384-91.
3. **Ciana P, Fumagalli M, Trincavelli ML, Verderio C, Rosa P, Lecca D, Ferrario S, Parravicini C, Capra V, Gelosa P, Guerrini U, Belcredito S, Cimino M, Sironi L, Tremoli E, Rovati GE, Martini C, Abbracchio MP.** The orphan receptor GPR17 identified as a new dual uracil nucleotides/cysteinyl-leukotrienes receptor. *Embo J*. 2006; 25: 4615-27.
